# Histopathological Analysis of Myocardial Remodeling Following Heart Failure: A Cadaveric Case Report

**DOI:** 10.7759/cureus.87889

**Published:** 2025-07-14

**Authors:** Anthony DeSomma, Jeung Woon Lee

**Affiliations:** 1 Anatomy, Lake Erie College of Osteopathic Medicine, Bradenton, USA; 2 School of Dental Medicine, Lake Erie College of Osteopathic Medicine, Bradenton, USA

**Keywords:** cardiac remodeling, cresyl violet, heart failure, histopathology, masson’s trichrome, myocardial fibrosis, post-mortem

## Abstract

Cardiovascular disease and aging are key contributors to myocardial remodeling, often leading to fibrosis, cellular hypertrophy, and impaired cardiac function. Understanding these structural changes is essential for explaining the pathophysiology of heart failure.

This study examined histopathological changes in myocardial tissue from a post-mortem cadaver with heart failure. Two age-matched cadavers without cardiac disease were used as controls. Ventricular tissue samples were thin-sectioned and stained for collagen deposition and cellular morphology using Masson’s Trichrome and Cresyl Violet dyes.

Histopathological findings showed the presence of increased collagen deposition and fibrosis in the myocardial tissue with heart failure, consistent with a stiffened myocardium. Additionally, Cresyl Violet staining showed cardiomyocyte hypertrophy compared to control samples, suggesting cellular remodeling, which is well characterized following heart failure.

These findings provide further histological evidence of myocardial remodeling in heart failure, characterized by fibrosis and cardiomyocyte hypertrophy. Further quantitative analysis is needed to deepen our understanding of these pathological changes and their implications for cardiac function.

## Introduction

Congestive heart failure (CHF) is a leading cause of morbidity and mortality worldwide, affecting an estimated 56.5 million individuals globally in 2021, a figure projected to rise significantly. Its prevalence is increasing steadily, in part attributable to the global aging population (1% among 45-55-year-olds vs. >10% over 80-year-olds) and improved survival rates from other cardiac-related diseases, such as myocardial infarction, allowing more individuals to live long enough to develop CHF [1-3].

Advanced heart failure represents a profound challenging clinical state with persistent and refractory symptoms despite optimal therapeutic interventions. This critical condition severely reduces patient well-being and places increasing demands on families, healthcare infrastructure, and the broader community [4]. To understand the multifaceted nature of heart failure, it is imperative to first understand its common etiologies, including hypertension, coronary artery disease, and specific conditions such as HIV-associated cardiomyopathy, all of which significantly contribute to its development and progression [5]. These underlying pathologies can lead to various myocardial alterations, often resulting in a restriction that results in diastolic dysfunction, thereby significantly impacting cardiac preload.

A hallmark of CHF is myocardial remodeling, a pathological process involving structural, functional, and cellular changes to the heart muscle in response to chronic stress and/or injury [6]. A distinctive feature of this remodeling is myocardial fibrosis, characterized by the excessive deposition of extracellular matrix proteins, particularly collagen type I and III, in the interstitium and the perivascular areas [7,8]. This fibrotic remodeling contributes to myocardial stiffening, impaired contractility, disrupted electrical conduction, and eventual cardiac decompensation by increasing collagen cross-linking and physically restricting the contraction and relaxation of myocytes [9,10].

Recent studies have shown that overexpression of CCL2 chemokines is associated with increased myocardial inflammation and fibrosis formation, and CCL2 knockout and anti-CCL2 antibody studies showed anti-fibrotic effects in animal studies. Additionally, plasma levels of the pro-inflammatory cytokines such as TNF-α, IL-1β, and IL-6 showed fibrinogenic activities associated with fibrosis formation [11].

Histopathological studies play a major role in understanding the cellular changes associated with myocardial remodeling. Among the commonly used staining techniques, Masson’s Trichrome stain is used to visualize collagen fibers, making it a good tool for identifying and quantifying fibrotic changes in cardiac tissue [12]. Cresyl Violet stain highlights cell nuclei and cytoplasmic components, allowing for the assessment of cellular architecture and myocyte density, which are often altered in the myocardium of CHF patients [5]. Prior research has identified that hearts affected by CHF may demonstrate an increased wall thickness, fibrosis, and reduced myocyte density, but direct post-mortem histological comparisons between CHF and non-CHF hearts are limited in the literature [13,14].

In this study, we investigated the structural changes in ventricular fibrosis, wall thickness, and myocyte density of left ventricular tissue obtained post-mortem from one individual with a history of CHF and two individuals without documented cardiovascular disease. By using Masson’s Trichrome and Cresyl Violet stains, this study aimed to better understand the cellular changes associated with myocardial remodeling in heart failure.

## Case presentation

A post-mortem histological comparison was conducted using left ventricular tissue from three human cadavers. The experimental sample was obtained from an 82-year-old woman with a documented history of CHF. Two control samples were obtained from individuals without a known history of cardiovascular disease: a 75-year-old woman who died from complications of cancer, and a 76-year-old man who died from pneumonia associated with COVID-19. Tissue samples were provided to the gross anatomy lab at the Lake Erie College of Osteopathic Medicine through a medical cadaver donation program.

A 2cm x 2cm transmural section of the left anterior ventricular wall was resected from each heart, ensuring consistency in the anatomical location. Each specimen was postfixed in a fresh 4% paraformaldehyde solution overnight and serially sectioned at 4 µm thickness using a freezing microtome (Microm HM450, Thermo Scientific, Waltham, MA). The tissue sections were mounted on gelatin-coated slides, dried overnight, and processed for Mason’s Trichrome and Cresyl Violet (ThermoFisher, Waltham, MA).

Cresyl Violet stain was used to assess myocyte density and cellular architecture, as it highlights neuronal and cytoplasmic structures. Masson’s Trichrome Stain was used to assess fibrosis, as it selectively stains collagen fibers blue, differentiating fibrotic tissue from muscle that stains red. The standard staining protocol was followed as suggested by the manufacturer (ThermoFisher, Waltham, MA).

Stained sections were digitally imaged using an inverted fluorescent microscope (Olympus IX51) at 4x magnification. Due to the size (area) of the tissue, histology images were digitally captured piece by piece as sequential tiles at both 4x and 20x magnifications. These tiles were subsequently combined using ImageJ (Fiji v.1.5.4) to reconstruct a full-section montage. Cell density was quantified using ImageJ software. The number of myocyte nuclei was counted in the epicardium, myocardium, and endocardium within two regions of interest (ROIs) per layer (dimensions: 1.47 mm × 1.10 mm). The counts were normalized to the area, and the values from all six regions were averaged. ROIs were chosen randomly, avoiding large vessels or artifacts. The threshold setting in Fiji was adjusted to highlight only the Cresyl Violet-stained myocyte nuclei. The density of myocytes was calculated as cells per mm². The wall thickness was measured in µm and averaged from 10 random locations across the left ventricular section encompassing the epicardium to the endocardium layers. Fibrosis was quantified by measuring blue-stained regions in Masson’s Trichrome sections using the color threshold tool in Fiji to isolate collagen fibers.

Given the study design, involving the detailed examination of tissue samples from a single case of CHF compared to two control cadavers, formal inferential statistical analyses were not performed. Instead, the quantitative results are presented descriptively as the total number of cell density, wall thickness, and transmural percentage of fibrosis, each normalized to a standardized tissue area per individual tissue sample.

Results

Left Ventricular Wall Thickness

**Figure 1:**
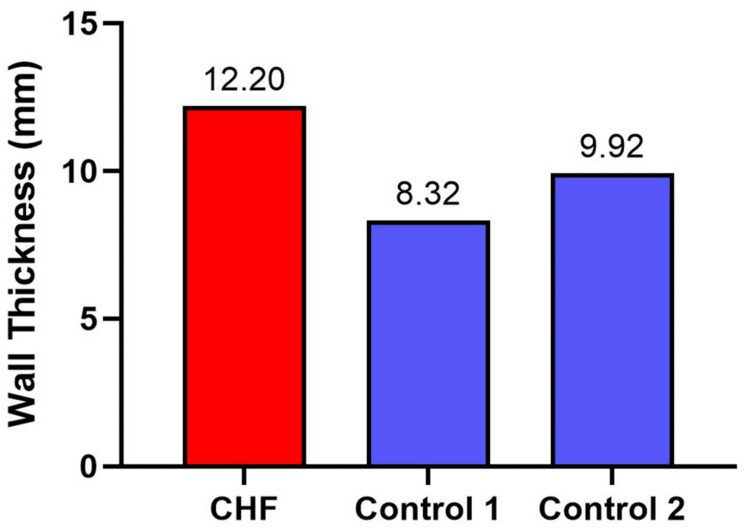
The left ventricular wall thickness from a patient with congestive heart failure (CHF) and two controls. The CHF heart exhibits an increased wall thickness compared to controls, consistent with pathological remodeling in heart failure.

Microscopic analysis of left ventricular cross-sections demonstrated a marked increase in the wall thickness in the experimental heart (12.20 mm) compared to the two control heart tissues (8.32 mm and 9.92 mm, respectively). These findings are consistent with left ventricular hypotrophy in CHF patients, an adaptation in response to chronic volume or ventricular pressure overload (Figure 1).

Myocardial Fibrosis

**Figure 2:**
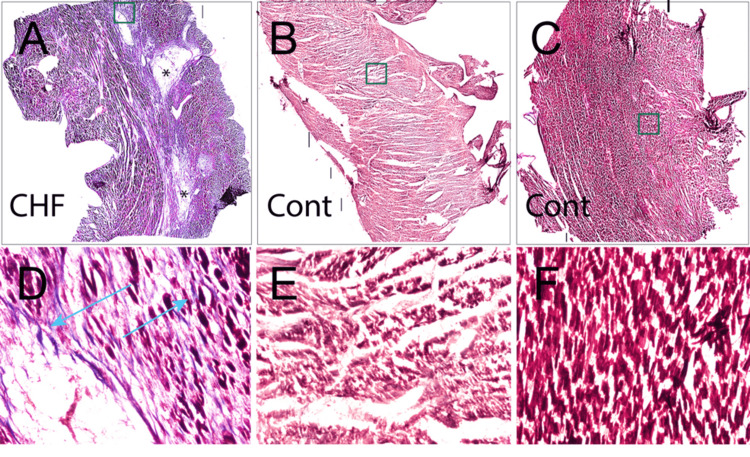
Composite histological sections of the left ventricular myocardium, prepared with Masson's Trichrome stain. Panels A-C show reconstructed whole-mount montages generated from 4x magnification image tiles, while panels D-F present single images captured at 4x magnification. The boxes in panels A, B, and C represent the regions where the high magnification images were derived (panels D, E, and F). The experimental tissue section showed diffuse fibrosis (blue stain; arrows) compared to control samples which showed preserved myocardial architecture and negligible fibrotic deposition. *: Scarred myocardial regions devoid of myocytes; CHF: congestive heart failure; Cont: control.

Histological staining with Masson’s Trichrome revealed an increase in fibrotic tissue in the CHF heart compared to controls (Figure 2).

**Figure 3:**
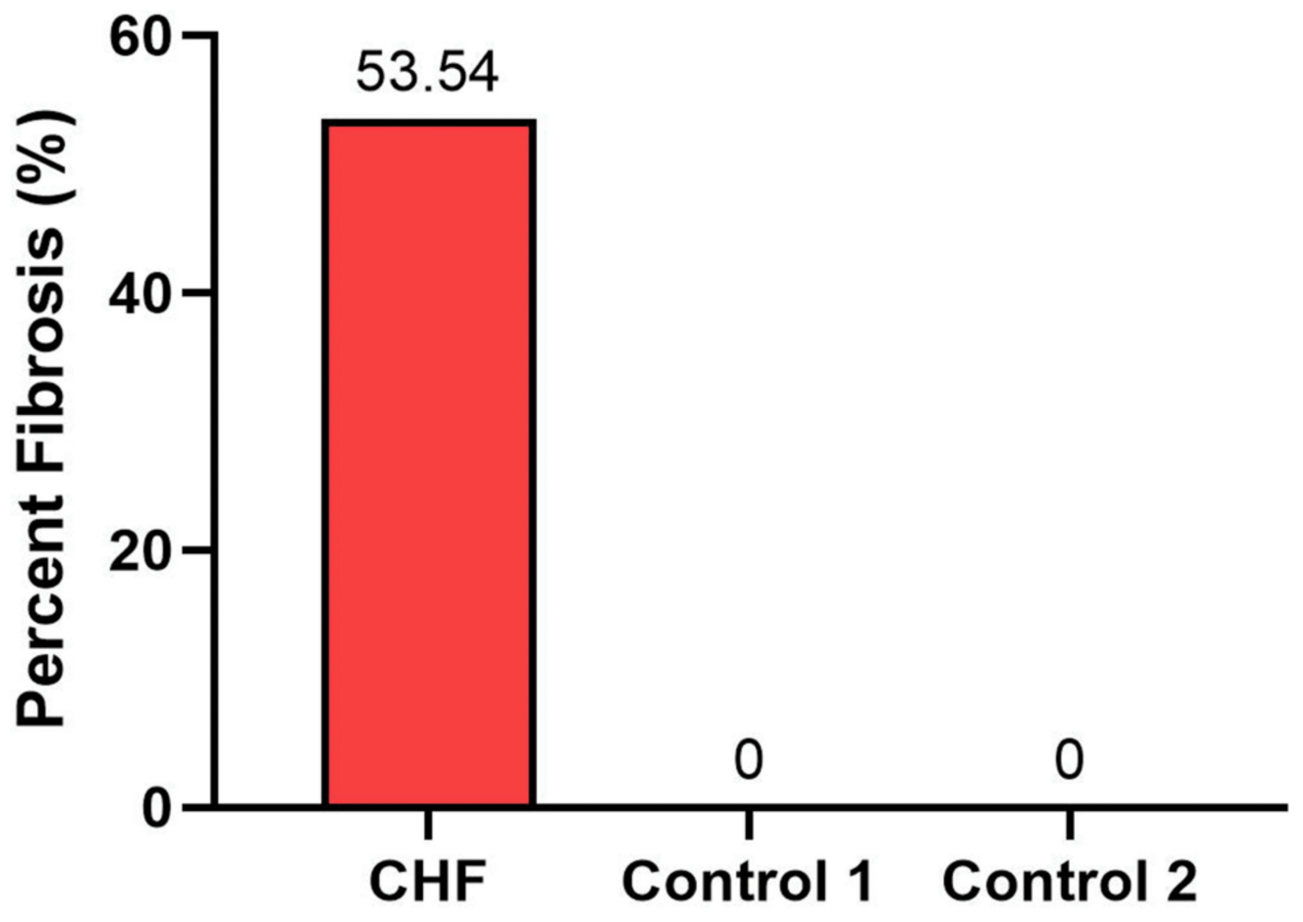
The percent myocardial fibrosis from a patient with congestive heart failure (CHF) and two controls. The CHF heart exhibited drastically elevated fibrosis compared to controls.

In the experimental tissue section, collagen fibers stained blue and were observed extensively in the interstitial and perivascular regions of the myocardium, with 250.78 mm^2^ of the fibrotic area detected from the 468.41 mm^2^ tissue area examined. This represented 53.54% of cardiac tissue area examined showing the presence of fibrosis. In contrast, the control specimens showed minimal to no collagen deposition from the similarly sized tissue area examined. These results demonstrate the pathological extracellular matrix remodeling associated with end-stage heart failure (Figure 3).

Myocyte Density

**Figure 4:**
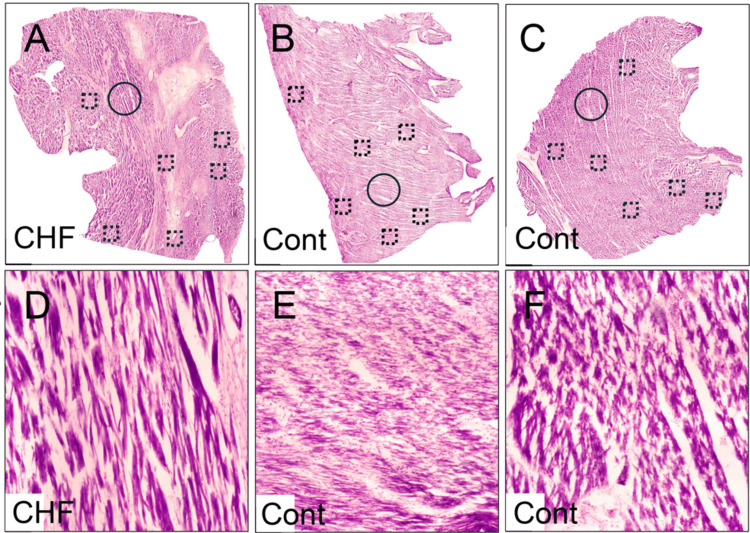
Composite histological sections of left ventricular myocardium stained with Cresyl Violet. Panels A-C show reconstructed whole-mount montages generated from 4x magnification image tiles, while panels D-F present single images captured at 4x magnification. The circles in panels A, B, and C represent the regions where the high-magnification images (panels D, E, and F) were derived. The boxed regions in panels A, B, and C indicate areas selected for cell density quantification. The CHF heart tissue at high magnification showed a decreased number of myocytes (low density) and disorganized architecture. The control tissues displayed uniformly arranged myocytes with a preserved structure. CHF: congestive heart failure; Cont: control.

Cresyl Violet staining showed a reduced number of myocardial cells in the experimental CHF tissue section. The myocardium of the CHF section showed increased spacing between and irregular arrangements of myocytes, likely reflecting apoptosis or necrosis due to chronic ischemia and remodeling (Figure 4).

**Figure 5:**
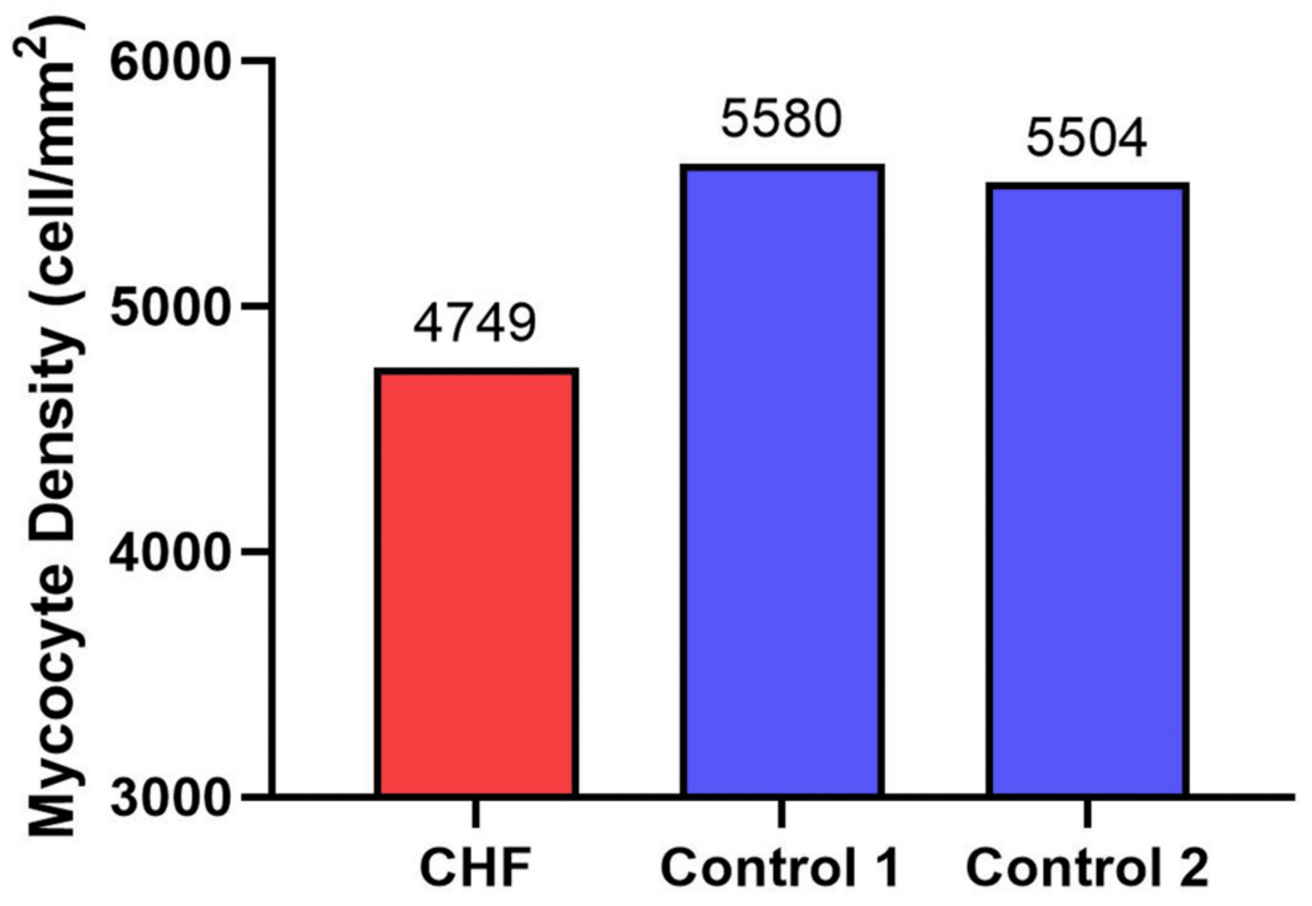
The number of ventricular myocytes from a patient with congestive heart failure (CHF) and two controls. The CHF heart displayed lower number of ventricular myocytes compared to controls.

Cresyl Violet staining revealed decreased myocyte density in the CHF heart compared to both controls, with the CHF heart having 4,749 cells/mm^2^, Control 1 having 5,580 cells/mm², and Control 2 having 5,504 cells/mm² (Figure 5).

## Discussion

Myocardial remodeling represents a critical pathological process in CHF, driving progressive cardiac dysfunction. This study examined the histopathological changes of the anterior ventricular wall from a post-mortem left ventricular tissue with a documented CHF, compared against age-matched controls, revealing a consistent pattern of structural and cellular alterations. Specifically, we observed a marked increase in wall thickness, extensive myocardial fibrosis, and a significant reduction in myocyte density. These findings further provide additional anatomical insights into the severity and nature of the complex remodeling processes that collectively contribute to impaired cardiac function in CHF.

The increased left ventricular wall thickness in our CHF tissue sample is consistent with previous reports associated with physiological response of cardiac hypertrophy, a compensatory mechanism against chronic pressure or volume overload [15,16]. While this hypertrophic remodeling initially serves to preserve cardiac output, its sustained presence ultimately transitions to a maladaptive state, contributing to augmented myocardial stiffness, increased myocardial oxygen demand, and subsequent cardiac decompensation [17,18].

The left ventricular wall thickness measured in our female CHF subject was marginally lower than the post-mortem values reported by Kitzman et al. (1988), who delineated pathological thickness as >1.5 mm and normal thickness within the range of 1.2 to 1.5 mm [16]. This minor discrepancy may be attributable to technical factors such as post-mortem fixation and tissue processing, a phenomenon supported by Lohner et al. (2025), who documented an increased ventricular wall thickness in post-mortem cardiac samples relative to ante-mortem measurements [15]. Additionally, this difference could be associated with the specific severity of CHF inherent to our cohort. Conversely, the left ventricular wall thickness observed in our control samples is in agreement with previously published data by Lohner et al. (2025) [15].

A notable finding in our analysis was the substantial increase in myocardial fibrosis within the CHF heart, where over 50% of the myocardial area displayed collagen fibers. This observation is in agreement with a previous report by Galati et al. (2016) who characterized myocardial fibrosis in end-stage hypertrophic cardiomyopathy hearts, reporting an overall range of 23.1% to 55.9% (mean 37.3% ± 8.4%) of the left ventricular myocardium [17]. Fibrosis disrupts the normal myocardial architecture, impairs contractility, and increases the risk of arrhythmias [18]. Previous studies have identified a strong correlation between the degree of myocardial fibrosis and adverse clinical outcomes in patients with CHF, reinforcing the importance of this histological marker in disease progression and prognosis [19,20].

In addition, we noted a reduction in myocyte density in the CHF heart. This may reflect progressive myocyte loss due to necrosis or apoptosis, a key feature of chronic heart failure due to oxidative stress, inflammation, and ischemia [21]. Olivetti et al. (1997) reported myocyte apoptosis in failing human hearts at an average rate of 2318 cells per 10^6 myocytes, which represents about a 232-fold increase in apoptosis compared to normal control hearts [22]. The increased spacing between myocytes observed in our Cresyl Violet-stained sections may further support this hypothesis and demonstrate the loss of myocytes and replacement with non-contractile fibrotic tissue. These findings align with previous literature describing the pathophysiological changes of heart failure in which mechanical stress promotes myocardial hypertrophy and fibrosis and further reduces cardiac efficiency and promotes further remodeling [23,24].

Several limitations are associated with this study, most notably the extremely small sample size, comprising a single CHF specimen and two controls. This inherently limits the generalizability of our findings. Also, although we selected controls of a similar age, confounding variables such as comorbidities and cause of death may have affected the myocardial structure. Lastly, while histological staining provides valuable structural information, we did not use immunohistochemical or molecular markers that could offer additional insights into specific remodeling pathways.

## Conclusions

This study highlights key histopathological features of myocardial remodeling in CHF, including an increased wall thickness, interstitial fibrosis, and reduced myocyte density, through direct, visual, and quantitative characterization of post-mortem human cardiac tissue. By methodically quantifying cardiac myocyte changes and the extent of fibrosis in CHF tissue compared to controls, this research underscores the utility of cadaver-based studies for both education and hypothesis generation in cardiac pathology. Future research with larger sample sizes and molecular analyses would be essential to further characterize the remodeling mechanisms and identify possible therapeutic targets for heart failure.
